# Habitat Heterogeneity Variably Influences Habitat Selection by Wild Herbivores in a Semi-Arid Tropical Savanna Ecosystem

**DOI:** 10.1371/journal.pone.0163084

**Published:** 2016-09-28

**Authors:** Victor K. Muposhi, Edson Gandiwa, Abel Chemura, Paul Bartels, Stanley M. Makuza, Tinaapi H. Madiri

**Affiliations:** 1 School of Wildlife, Ecology and Conservation, Chinhoyi University of Technology, Private Bag 7724, Chinhoyi, Zimbabwe; 2 School of Agricultural Sciences and Technology, Chinhoyi University of Technology, Private Bag 7724, Chinhoyi, Zimbabwe; 3 Department of Nature Conservation, Tshwane University of Technology, Private Bag X680, Pretoria, 0001, South Africa; 4 Zimbabwe Parks and Wildlife Management Authority, P.O. Box CY140, Causeway, Harare, Zimbabwe; Sichuan University, CHINA

## Abstract

An understanding of the habitat selection patterns by wild herbivores is critical for adaptive management, particularly towards ecosystem management and wildlife conservation in semi arid savanna ecosystems. We tested the following predictions: (i) surface water availability, habitat quality and human presence have a strong influence on the spatial distribution of wild herbivores in the dry season, (ii) habitat suitability for large herbivores would be higher compared to medium-sized herbivores in the dry season, and (iii) spatial extent of suitable habitats for wild herbivores will be different between years, i.e., 2006 and 2010, in Matetsi Safari Area, Zimbabwe. MaxEnt modeling was done to determine the habitat suitability of large herbivores and medium-sized herbivores. MaxEnt modeling of habitat suitability for large herbivores using the environmental variables was successful for the selected species in 2006 and 2010, except for elephant (*Loxodonta africana*) for the year 2010. Overall, large herbivores probability of occurrence was mostly influenced by distance from rivers. Distance from roads influenced much of the variability in the probability of occurrence of medium-sized herbivores. The overall predicted area for large and medium-sized herbivores was not different. Large herbivores may not necessarily utilize larger habitat patches over medium-sized herbivores due to the habitat homogenizing effect of water provisioning. Effect of surface water availability, proximity to riverine ecosystems and roads on habitat suitability of large and medium-sized herbivores in the dry season was highly variable thus could change from one year to another. We recommend adaptive management initiatives aimed at ensuring dynamic water supply in protected areas through temporal closure and or opening of water points to promote heterogeneity of wildlife habitats.

## Introduction

Understanding the processes driving habitat selection and or the suitability of habitat patches by wild herbivores is essential in conservation biology as it provides insights into the processes driving population dynamics, community structure and functioning in ecosystems [1, 2]. In this regard, habitat selection theory has greatly influenced the understanding of dispersal, source-sink dynamics, occupation and avoidance of ecological traps in natural ecosystems [3]. Habitat preferences by wild herbivores are assumed to be adaptive such that the use of preferred habitats increases fitness [4]. Habitat selection theory suggests that habitat preference by wild herbivores is meant to increase their fitness, however, the suitability of preferred habitats would decline with increasing population density and increased competition [5]. Accordingly, habitat selection by wild herbivores will exhibit increasing preference for areas with desirable microclimate and dietary requirements of wildlife species [6].

Temporal and spatial variability in habitat suitability may be influenced by several factors ranging from physical ecosystem modifications or engineering to climate variability [2, 7]. These dynamics promotes heterogeneity in ecosystems that ultimately may render them suitable or less suitable for wild herbivores [8]. Studies have shown that in most ecosystems, habitat suitability and selection is driven by the following: habitat quality [9, 10], surface-water availability [11, 12], competition [13], perceived hunting or predation risk and disturbances [14, 15].In human-mediated environments habitat selection by wild herbivores is indirectly influenced by such factors as habitat loss and fragmentation [16, 17], illegal harvesting [18, 19], unsustainable utilization of wildlife resources [20, 21], fires[22–24]and droughts [25, 26]. In most savanna ecosystems, fire has been observed to influence vegetation cover thus affecting the suitability of habitat patches subjected to unplanned and frequent fires [24, 27].

Similarly, the allometry of herbivore feeding due to the different anatomical and physiological adaptations has been hypothesized to influence habitat selection by herbivores [28]. The Bell-Jarman principle asserts that large herbivores have a wider food quality tolerance than medium or smaller herbivores [29, 30]. Due to greater digestive efficiency and lower metabolic demands per unit body mass, large herbivores can survive on a diet of lower nutritional value than can small herbivore species [28]. The principle further shows that smaller herbivores can survive in habitats where food plants are in low abundance, because of lower total metabolic demand [31]. In contrast, large herbivores have a wider feed quality base that enables them to select diverse habitat types and as such, may utilize a higher proportion of the landscape compared to medium or smaller herbivore species [8]. However, in some protected areas where selective harvesting (i.e., trophy hunting) is allowed through regulated offtake levels [32], wild herbivores may select poor habitat patches as a way of avoiding human disturbances or exposure to hunting risk [33, 34]. The magnitude of management interventions in some of these ecosystems, e.g., artificial surface water provisioning may reduce habitat heterogeneity and result in the alteration of herbivore species distribution patterns [35, 36]. Extensive road and trail networks have been also reported to influence the distribution patterns of wild herbivores in some ecosystems [34, 37, 38].

In tropical savanna ecosystems, few studies have attempted to explore the spatial distribution of wild herbivores in protected areas falling under the International Union for Conservation of Nature and Natural Resources (IUCN) Category VI, which are managed mainly for the sustainable use of natural ecosystems and as buffer zones surrounding national parks [39]. However, most of the studies have been conducted in national parks in the absence of legal hunting activities, e.g., South Africa and Zimbabwe [2, 8, 11, 40]. In these previous studies, species distributions have been reported to be influenced by environmental factors such as surface water availability, vegetation cover and anthropogenic disturbances such as roads and hunting activities [41, 42]. However, due to avoidance mechanisms to evade human disturbances, wild herbivores have been observed to avoid areas with elevated hunting pressure in favor of those with minimum exposure to hunting [33, 43].

Since habitat heterogeneity is a key driver of herbivore spatial distribution in natural ecosystems, it is likely that the occurrence of herbivore species in human-mediated environments, particularly a hunting area, may be affected by vegetation cover (i.e., a measure of habitat quality in the form of cover, shade and feed) and availability of surface water as well as human disturbances associated with hunting activities. Considering that managers can only protect species whose distributional information is known [44], an understanding of species compositional and spatial distribution patterns information and the factors that influence them is essential for sustainable wildlife conservation planning and management in human-mediated environments [45]. Such information is also considered essential for mapping spatial distribution of priority areas for conservation and the enactment of sound conservation policies integral in sustainable protected area management [46, 47].

We modeled the spatial distribution of four wild herbivores, i.e., two large herbivores, namely African elephant, Cape buffalo (*Syncerous caffer*),and two medium-sized herbivores, namely, sable (*Hippotragus niger*) and greater kudu (*Tragelaphus strepsiceros*) in relation to surface water availability, vegetation cover and roads in Matetsi Safari Area, northwest Zimbabwe for the years 2006 and 2010. Specifically, we predicted that: (i) surface water availability, vegetation cover and roads have a strong influence on the spatial distribution of wild herbivores in the dry season, (ii) area of suitable habitats for large herbivores would be greater compared to medium sized herbivores, and (iii) temporal spatial extent of suitable habitats for wild herbivores would be highly variable.

## Materials and Methods

Permission to conduct the study in Matetsi Safari Area (which is a protected area) was obtained from Zimbabwe Parks and Wildlife Management Authority, which is the relevant regulatory body responsible for Parks Estates in Zimbabwe. PERMIT NO.: 23 (1) (C) (II) 73/2015.The sampling procedures for this study were considered as part of the field permit application process.

### Study Area

**Fig 1:**
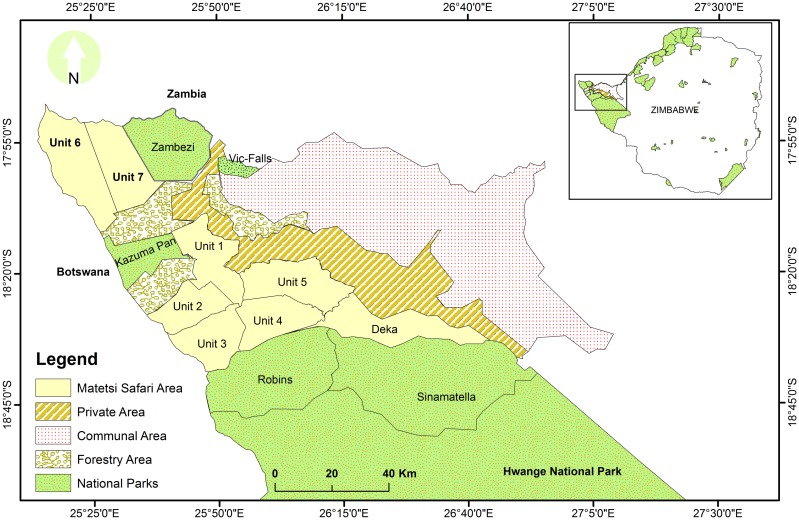
Location of study area, Matetsi Safari Area and the surrounding areas (i.e., National Parks, Forestry Areas, Private Areas and Communal Areas in northwest Zimbabwe). Insert: Location of study area (solid rectangle) in Zimbabwe in relation to other protected areas. Notes: Private area is considered as an Environmental Conservation Area (ECA), has a mixed land use which integrates both wildlife conservation and arable agriculture. Source: shape files based on the Surveyor General’s maps of Zimbabwe.

The study was conducted in Matetsi Safari Area with an approximate area of 3,000 km^2^, between 25820’–26830’ longitude and 18810’–18845’ latitude, north-western Zimbabwe (Fig 1). Matetsi Safari Area is part of the Kavango Zambezi Transfrontier Conservation Area (KAZA TFCA) which is shared between Angola, Botswana, Namibia, Zambia and Zimbabwe established in 2011 [48]. Matetsi Safari Area is divided into seven management blocks called Units (Table 1).

**Table 1:** Characteristics of the seven management Units, (i.e., hunting status and estimated area), of Matetsi Safari Area, Zimbabwe.

Unit	Concession holder	Hunting status	Area (km^2^)
1	*Private concession	Since 1973	398
2	††ZimParks	since 2013	475
3	Private concession	Since 1973	293
4	ZimParks	Since 2012	358
5	ZimParks	Since 2005	364
6	Private concession	Since 1973	592
7	Private concession	Non hunting	447

Notes:

^††^ZimParks stands for Zimbabwe Parks and Wildlife Management Authority.

*Private concession here refers to a medium to long-term lease given to a private outfitter by the Zimbabwe Parks and Wildlife Management Authority to conduct hunts in a Safari Area within the Parks and Wildlife Estate.

The southern block (i.e., comprises of Unit 1–5) is bordered by Hwange National Park to the south, north-eastern side with private and communal areas whereas the western side is mostly Kazuma Pan National Park and Forestry Hunting Area. However, the northern block (i.e. Unit 6 and 7), is sandwiched by protected areas, i.e., Zambezi National Park to the eastern side, and to the western side are Chobe National Park in Botswana and Forestry Area to the south. Trophy hunting has been the sole land use option for Matetsi Safari Area for more than 37 years [49]. Of these six management blocks, only Unit 7 is reserved for photographic tourism and without any form of legal hunting allowed therein. The mean annual rainfall for the study area is 645mm per annum received between November and March whilst the long-term average rainfall for the study area is 606mm [50]. Temperature range from 19.8°C in July to 32.6°C in October [33]. The area occurs in a low rainfall region and is characterized by deep Kalahari sands. This makes natural surface water scarce during the dry season as most rivers except for a few river pools which retain water throughout the year but these mostly occurs within Robins Camp of Hwange National Park [11]. Wildlife species are able to meet their water requirements during the dry months of the year (May to October) from artificial water holes that are replenished through pumping of ground water to create a year round surface water supply in Matetsi Safari Area and its neighboring Hwange National Park. However, artificial water holes are unevenly distributed across the study area.

The main soil types on sites are lithosols and regosols occurring on Karoo volcanic and Kalahari geological formations, respectively [51]. The lithosols are dominated by *Colophospermum mopane* and *Terminalia* species[52], whilst *Baikiaea plurijuga*, which occurs in association with *Pterocarpus angolensis* and *Guibortia coleosperma* dominate on the regosols [51]. Some of the common wildlife species in the study area include: herbivores (Cape buffalo, Burchell’s zebra (*Equus quagga*), elephant, giraffe (*Giraffa camelopardalis*), greater kudu, impala (*Aepyceros melampus*), reedbuck (*Redunca arundinum*), sable antelope, warthog (*Phacochoerus aethiopicus*) waterbuck (*Kobus ellipsiprymnus*), wildebeest (*Connochaetes taurinus*)) and carnivores (leopard (*Panthera pandus*), African lion (*Panthera leo*), and spotted hyena (*Crocuta crocuta*)).

### Study species

We selected four wild herbivores as study species: two large herbivores (African elephant and Cape buffalo) and two mid-sized herbivores (greater kudu and sable). These four species were selected on the basis that they are amongst the most commonly legally hunted herbivores in the study area as well as in southern Africa [53, 54]. The densities of these species in this area: African elephant (0.7 individuals per km^2^), Cape buffalo (1.4 individuals per km^2^), sable (0.7 individuals per km^2^) and greater kudu (1.4 individuals per km^2^) for the period 1995–2010 as obtained from road strip counts data[55].

### Species occurrence data and environmental predictors

Species presence-only data for Unit 1–6 were obtained from Matetsi Safari Area Headquarters for the year 2006 and 2010 based on ensemble data collected using road strip counts as part of long-term systematic monitoring programs of the area for adaptive management (S1 File). Road strip counts data are one of the conventional and reliable methods used in most savanna ecosystem to determine distribution and abundance of wildlife species [56, 57]. These surveys were conducted during the dry season between August and October when visibility was optimal.

Vegetation cover was estimated from the computed normalized difference vegetation index (NDVI) from available SPOT S10 hypertemporal NDVI data. The SPOT S10 NDVI dataset consists of 10-day maximum NDVI value composites from the SPOT VEGETATION 1 and VEGETATION 2 instruments on board SPOT 4 and SPOT 5 Satellites respectively. SPOT NDVI data was downloaded from the Flemish Institute for Technological Research (VITO) Product Distribution Portal (http://www.vito-eodata.be). These data have a spatial resolution of 1 km and temporal resolution of 10 days and come as digital numbers (DN values). To convert the DN values to NDVI, we used the formulae: NDVI = (0.004*DN value)-0.1(http://www.spot-vegetation.com). NDVI raster files were re-sampled from 1km to 600m using the resample (data management) tool in ArcGIS 10.2.1 (Environmental Systems Research Institute: Redlands, California, USA). NDVI is an effective estimate of vegetation productivity and standing biomass in semiarid systems [11]. NDVI can be used as a proxy for vegetation cover where high values represent green and nutritive vegetation whereas low values represent dry vegetation with low nutritive value [58]. In this study, NDVI was used to indicate vegetation cover, considered as a proxy for habitat quality (cover, space and feed).

**Fig 2:**
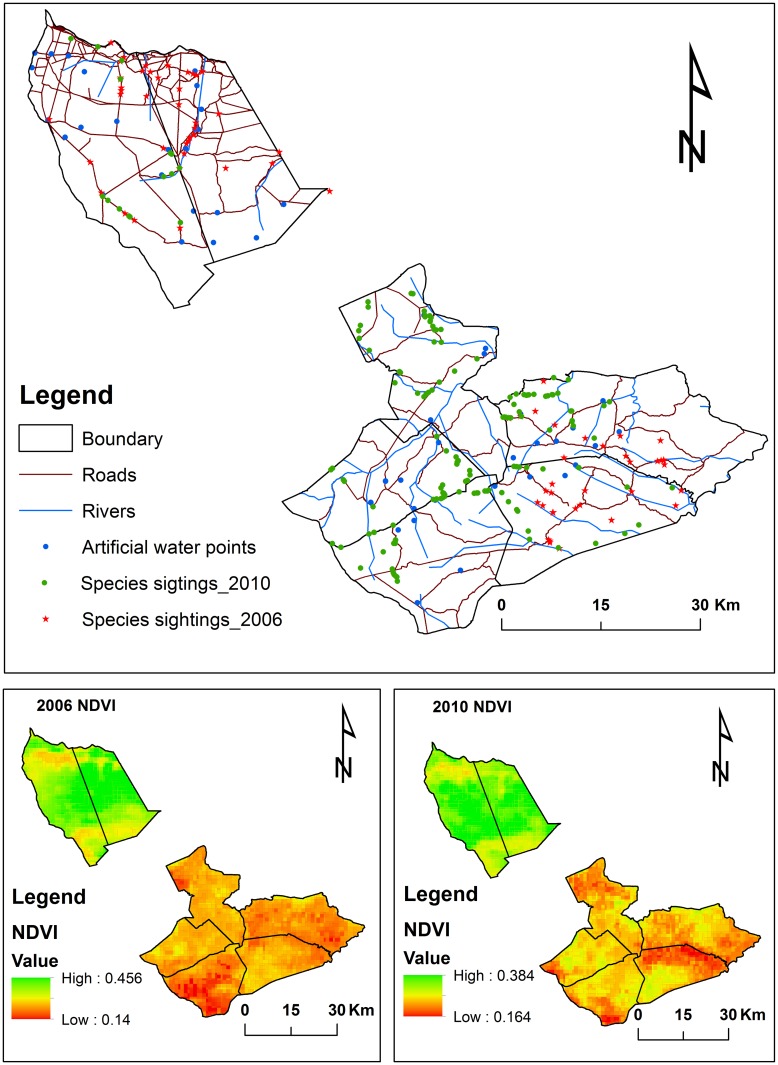
Environmental variables (roads, rivers, artificial water points and NDVI) used in the modeling of the habitat selection by four wild herbivores in Matetsi Safari Area, Zimbabwe.

Using the Euclidean distance algorithm in ArcGIS, distance of locations from the nearest artificial water point (D_w_), river (D_r_) and roads (D_rd_) were calculated. The location of the artificial water points were obtained from Matetsi Safari Area monitoring database of functional water holes for 2006 and 2010. The geographic locations of artificial water holes were imported into ArcGIS to create shape files for the surface water distribution in Matetsi Safari Area. Given that rivers tend to create microhabitats which are often preferred by most species during the dry season even when they have dried up, we incorporated rivers as an environmental predictor. This preference is mostly due to the availability of fairly palatable forage during drier months of the year compared to other habitat patches [59]. We checked if the environmental variables were not conflating using the Variance Inflation Factor (VIF) and found that there variables were not confounding as all of the correlations had VIF less than one (S1 Table). Species distribution based modeling has a common problem of spatial autocorrelation[60]. To address this problem, we computed the Moran’s I under spatial statistics tool in ArcGIS. From the spatial autocorrelation report (Moran’s Index of -0.34, z-score of -1.278, p = 0.202), we concluded that the pattern of observations (presence only data) seemed to be random and not clustered. All the data sets for the environmental variables were exported as ASCII files for the maximum entropy modeling, using MaxEnt software version 3.3.3k [61]. The environmental factors used in the modeling are shown in Fig 2.

### Spatial distribution modeling of wild herbivores

Maximum entropy (MaxEnt) approach[61]was used to model the spatial distribution of wild herbivores in relation to environmental factors in Matetsi Safari Area. We chose MaxEnt modeling because it is considered as an efficient and robust model for predicting species distributions from presence-only species data [62] and it can handle complex interactions between response and predictor variables yet sensitive to small sample sizes [63, 64]. MaxEnt is robust because even with small samples (i.e. from road counts), it is able to provide useful information in modeling suitable habitats [65]. The MaxEnt modeling approach is also robust because it incorporates both statistical models and machine learning techniques for modeling the occurrence of species [66, 67]. In most extensive protected areas where systematic wildlife surveys tend to be sparse, irregular and limited in proportion of area coverage particularly in sub-Saharan Africa, MaxEnt modeling is considered to be more suitable [41, 61]. The detailed information and description of MaxEnt is outlined elsewhere [61, 62, 68].

Using the MaxEnt technique, we built two separate models (i.e., for 2006 and 2010) predicting the spatial distribution of four wild herbivores, (i.e., two large herbivores: African elephant and Cape buffalo; and two mid-sized herbivores: greater kudu and sable) based on NDVI, distance from roads, artificial water points and rivers. We used the default variable response settings in MaxEnt to run the model [63], (500 maximum iterations, maximum number of background points of 10^5^, convergence threshold of 10^−5^ and maximum iteration value of 1000). We used the Bias file function in MaxEnt to adjust for sampling bias which is usually associated with the presence only data obtained from the road counts.

To evaluate the predictive ability of two MaxEnt models, we used the Area Under Curve (AUC) of the Receiver Operating Characteristic (ROC) curve technique [61, 69]. This technique, i.e., ROC (AUC) is the most important index of model quality because it provides an overall single measure of model accuracy that is not dependent upon a selected threshold [70]. Response curves for both MaxEnt models were used to test the response of the four wild herbivores to NDVI, distance to roads, artificial water points and rivers. The rule of thumb is that if the AUC is less than or equal to 0.5, the prediction of the model is random whilst for AUC greater than 0.5, the prediction is more than random, as attributed to environmental variables used in the model [71]. The logistic threshold of equal training and test sensitivity was used to produce a binary map showing the spatial extent of potential suitable and unsuitable habitats [72], for the four wild herbivores for 2006 and 2010. The logistic threshold of equal training and test sensitivity method was selected because it balances the accuracy of areas correctly modeled as present as well as those correctly modeled as absent in the training and test data [73]. We imported the predicted binary map into ArcGIS for proper representation and determination of areal extent of the predicted distribution using the area calculation algorithm [58].

The proportion of the predicted area (%) under suitable habitats for Cape buffalo and African elephant were pooled together to represent the suitable habitats for large herbivores whilst that of greater kudu and sable were pooled for medium-sized herbivores. We later used the predicted suitable area for large herbivores and medium-sized herbivores for the year 2006 and 2010 to compute Mann-Whitney tests to: (1) establish if area under suitable habitats for large herbivores was greater compared to medium-sized herbivores, and (2) determine if there was a difference in the area of suitable habitats for large and medium sized herbivores between the year 2006 and 2010 using Origin Pro 9software (OriginLab, Northampton, MA).

## Results

### Large herbivores probability distribution

**Fig 3:**
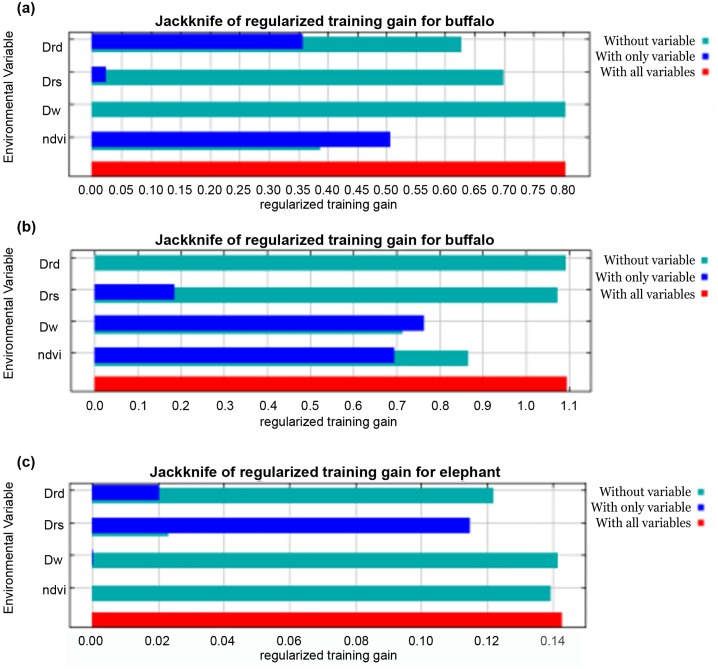
MaxEnt model showing the Jackknife test of the importance of variables used in training the distribution model of Cape buffalo, (A) year 2006, (B) year 2010, and African elephant (C) year 2006. Notes: Drd—Distance from roads, Drs—distance from rivers, Dw—distance from artificial water points, ndvi—Normalized Difference Vegetation Index.

MaxEnt modeling using NDVI, D_w_, D_rs_ and D_rd_ successfully explained the spatial distribution of Cape buffalo for 2006 (AUC = 0.913) and 2010 (AUC = 0.941). In 2006, NDVI explained much of the variation (57.7%) in the model (Fig 3A and Table 2) whilst in 2010, distance from artificial water points accounted for much of the variation (59.5%) in the model (Fig 3B). The variation in the probability of occurrence of African elephants in 2006 (AUC = 0.776) was explained by NDVI, D_w_, D_rs_ and D_rd_ as determined through MaxEnt modeling (Fig 3C). Distance from the rivers was the most significant variable accounting for 81.5% in the model variation in 2006 (Table 2). However, the MaxEnt modeling using NDVI, D_w_, D_rs_ and D_rd_ could not successfully explain for the variability in African elephant occurrence in for year 2010 (AUC = 0.500). This shows that in 2010, the environmental variables used in the model could not account for the distribution of African elephants.

**Table 2:** Relative contribution of the environmental variables used in MaxEnt modeling of habitat selection by four wild herbivores for the year 2006 and 2010 in Matetsi Safari Area, northwest Zimbabwe.

Variable	% contribution		Permutation importance	
	2006	2010	2006	2010
**Cape buffalo**				
NDVI	57.70	36.70	35.80	60.90
Distance from artificial water points (m)	0.00	59.50	0.00	28.80
Distance from rivers (m)	11.70	3.80	16.60	10.40
Distance from roads (m)	30.60	0.00	47.60	0.00
**African elephant**				
NDVI	1.70	*	8.30	*
Distance from artificial water points (m)	0.60	*	2.60	*
Distance from rivers (m)	81.50	*	65.20	*
Distance from roads (m)	16.20	*	23.90	*
**Greater kudu**				
NDVI	5.00	51.50	10.90	47.10
Distance from artificial water points (m)	7.40	31.50	4.30	22.70
Distance from rivers (m)	21.80	4.30	21.20	12.60
Distance from roads (m)	65.80	12.70	63.60	17.50
**Sable**				
NDVI	19.00	2.10	24.40	14.00
Distance from artificial water points (m)	2.50	61.30	1.50	41.40
Distance from rivers (m)	21.50	2.10	19.40	14.00
Distance from roads (m)	51.10	35.20	54.60	40.00

*The suitability model for elephant did not successfully explain the probability of occurrence of elephant in 2010

**Fig 4:**
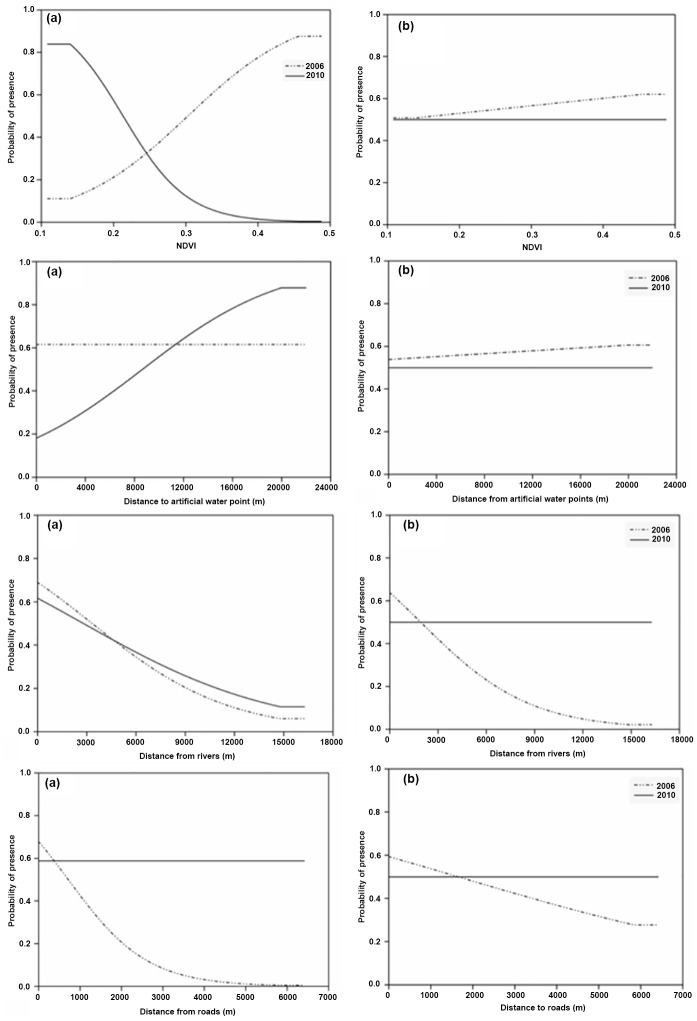
Response curves derived from MaxEnt Models showing influence of environmental variables on probability of occurrence of large herbivores, (A) Cape buffalo, and (B) African elephant for the year 2006 and 2010 in Matetsi Safari Area, Zimbabwe.

The probability of occurrence of Cape buffalo was positively correlated with NDVI in 2006 whilst in 2010, buffalo probability of occurrence declined with increasing NDVI (Fig 4A). Our results show that probability of occurrence of Cape buffalo declined with increasing distance from rivers for both 2006 and 2010. However, there was much variation in the effects of the four studied environmental variables on the probability of occurrence of Cape buffalo.

**Fig 5:**
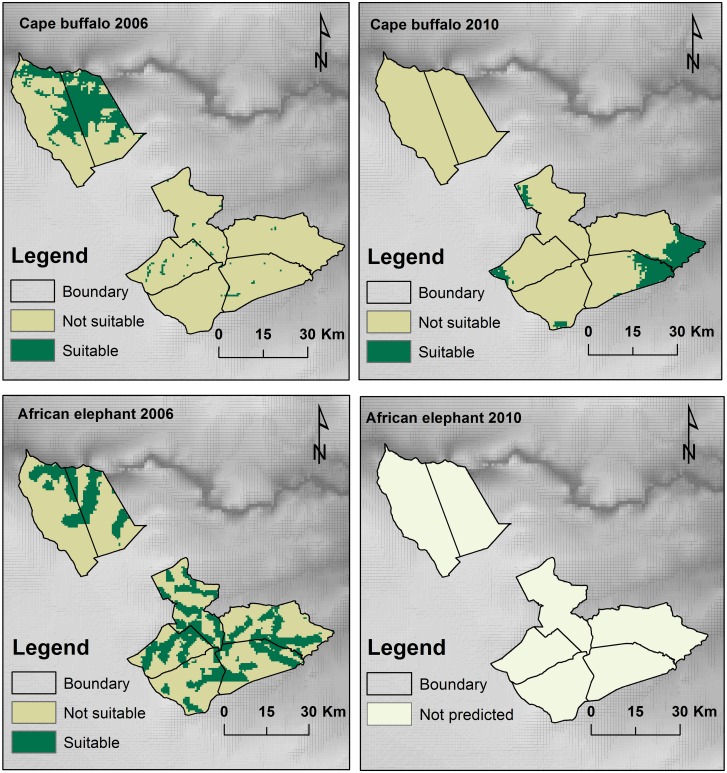
MaxEnt models showing the probability distribution of two large herbivores, Cape buffalo and African elephant for the year 2006 and 2010 in Matetsi Safari Area.

Our results show that distance from rivers accounted for much of the variation in the probability distribution of African elephants for 2006 (Table 2 and Fig 5). Both Cape buffalo and African elephant were not widely distributed but exhibited some degree of aggregation in certain sections of the study area.

### Medium-sized herbivores probability distribution

**Fig 6:**
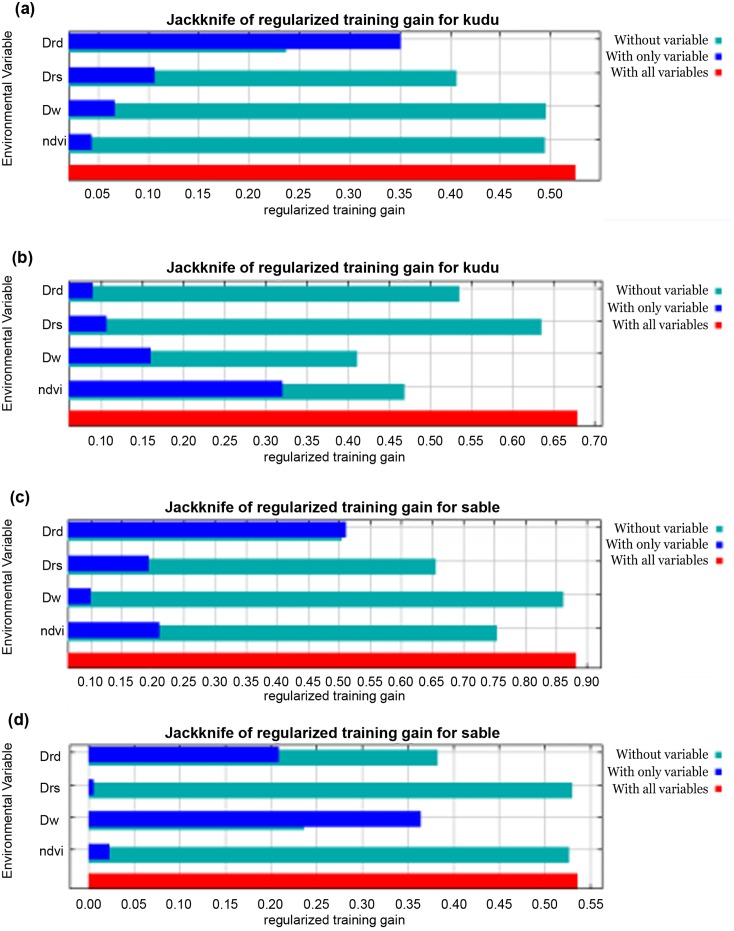
MaxEnt model showing the Jackknife test of the importance of variables used in training the distribution model for (A) greater kudu in 2006, (B) greater kudu in 2010, (C) sable in 2006, and (D) sable in 2010. Notes: Drd—Distance from roads, Drs—distance from rivers, Dw—distance from artificial water points, ndvi—Normalized Difference Vegetation Index.

The probability of occurrence of Greater kudu was successfully explained by NDVI, D_w_, D_rs_ and D_rd_ in 2006 (AUC = 0.799) and 2010 (AUC = 0.840) through MaxEnt modeling. In 2006, much of the variability in the model was accounted for by distance from roads (65.8%, Table 2). However, in 2010, NDVI contributed 51.5% of the variability in Greater kudu probability of occurrence (Fig 6A and 6B). Similarly, the four studied environmental variables had a good predictive power of MaxEnt modeling on the probability distribution of sable for the year 2006 (AUC = 0.900) and 2010 (AUC = 0.830). Distance from roads explained much of the variation in the model with a percentage contribution of 57.1% (Fig 6C and 6D).

**Fig 7:**
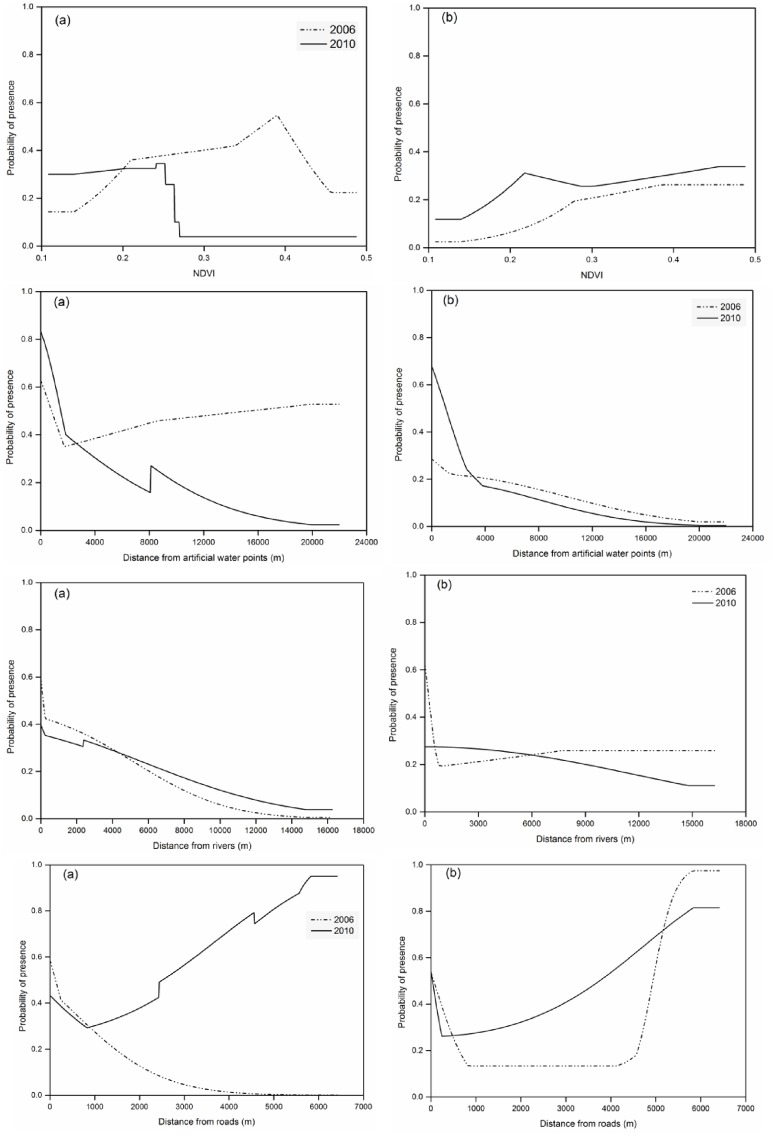
Response curves derived from MaxEnt Models showing influence of environmental variables on probability of occurrence of medium herbivores, (A) greater kudu, and (B) sable for the year 2006 and 2010 in Matetsi Safari Area, Zimbabwe.

**Fig 8:**
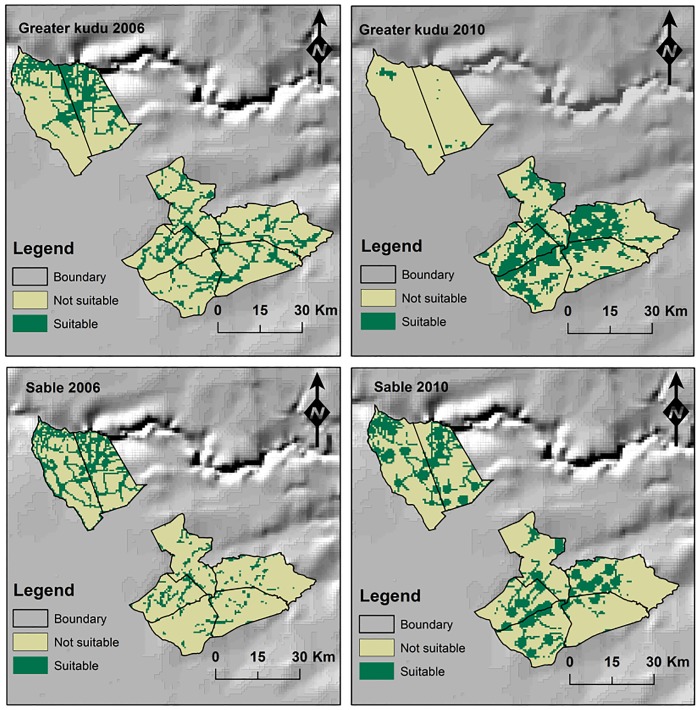
MaxEnt models derived probability distribution of greater kudu and sable for the year 2006 and 2010 in Matetsi Safari Area, Zimbabwe.

Generally, the probability of occurrence of greater kudu declined with increasing NDVI, distance from artificial water points and distance from rivers. However, greater kudu probability distribution increased with increasing distance from the roads in 2006 (Fig 7A). Overall, the probability of occurrence of sable increased with increasing distance from roads for both 2006 and 2010. However, the modeled probability of occurrence of sable declined with increasing distance from artificial water points indicating that they mostly occur in areas close to watering points (Fig 7B). There were variations in the modeled probability distribution of greater kudu and sable for the year 2006 and 2010 as influenced by the four environmental variables (Fig 8).

**Fig 9:**
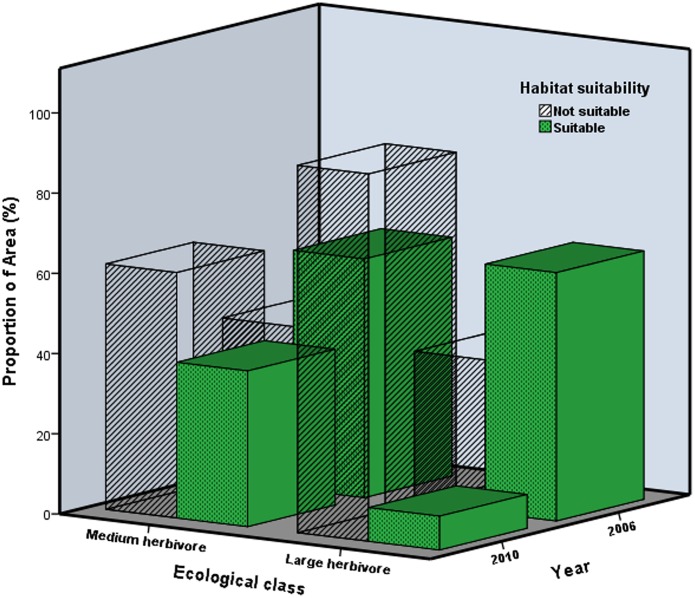
Proportion of predicted suitable and not suitable area for medium and large herbivores for the years 2006 and 2010 in Matetsi Safari Area, Zimbabwe.

**Fig 10:**
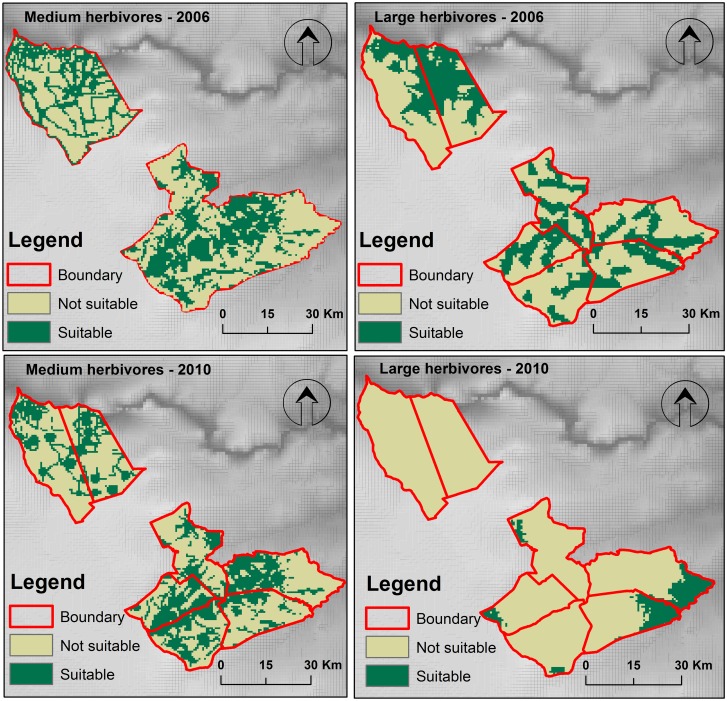
Probability distribution models of medium and large sized herbivores for the year 2006 and 2010 in Matetsi Safari Area, Zimbabwe derived from MaxEnt modeling.

Though there was a spatial variability in the habitat suitability of large and medium-sized herbivores, the overall predicted suitable habitat for the year 2006 and 2010 of large herbivores (median = 409.68 km^2^, range = 680 km^2^) and medium-sized herbivores (median = 727.38 km^2^, range = 189 km^2^, Table 3) in Matetsi Safari Area were not different (Mann-Whitney U test: U = 8.00, Z = 0.02, p = 0.987). There was no difference in the area suitable for large and medium-sized herbivores between 2006 and 2010 (Mann-Whitney U test: U = 6.00, Z = -0.58, p = 0.564). However, there was a variation (p < 0.000) in the proportion of suitable area and not suitable area for medium and large herbivores in 2006 and 2010 (Fig 9). There was an overlap of approximately 21.18% of the suitable habitat for both medium and large herbivores in the year 2006. However, in the year 2010, the overlap predicted suitable habitat for medium and large herbivore increased to 45.78% (Fig 10).

**Table 3:** Predicted total area of suitable habitats for the four wild herbivores for 2006 and 2010 in Matetsi Safari Area, Zimbabwe obtained through MaxEnt modeling.

Species	Year 2006		Year 2010		% change in suitable area
	More suitable area (km^2^)	% of total area	More suitable area (km^2^)	% of total area	
Cape buffalo	409.68	14.18	241.56	8.36	-41.00
African elephant	921.60	31.89	*	*	*
Greater kudu	722.52	25.00	732.24	25.34	1.00
Sable	598.68	20.72	787.32	27.25	32.00

*The suitability change for elephant was not calculated because the model did not successfully explain the probability of occurrence of elephant in 2010

## Discussion

We predicted that surface water availability, vegetation cover and human presence would have a strong influence on the spatial distribution of wild herbivores in the dry season for both large and medium-sized herbivores. Our results show that buffalo habitat suitability was mostly influenced by vegetation cover (i.e., NDVI) and distance from rivers. Though most rivers in the study area are ephemeral [11], the unique vegetation structure and composition attributes of the riverine ecosystem during the dry season would offer the most suitable habitat for the animals to meet their habitat requirements other than water [74]. In this study, buffalo probability of occurrence was higher in areas closer to rivers concurs with the assertions that buffalo are riverine species preferring to aggregate along rivers in the drier periods of the year. Riverine ecosystems are mostly preferred by buffalo because they provide good quality and quantity feed, shade and cover from predation during the dry season [59, 75, 76].

Contrary to the results in 2006, we found buffalo to prefer habitat patches characterized with higher NDVI values in 2010. This contrasting temporal shift in the habitat selection by buffalo may be due to water related constrains as in 2010, buffalo mostly selected areas closer to artificial water points. Due to the piosphere effect, vegetation cover surrounding artificial water points has been reported to be low [77]. It is likely that when water is limiting during drier periods of the year, buffalo may prefer to utilize habitat patches close to a water source regardless of the feed quality associated with such habitats [75]. Though NDVI, is a measure of habitat quality (i.e. feed availability, both quality and quantity, and shade), human disturbances may cause animals to avoid certain areas in favor of poor habitat patches[78]. This avoidance strategy results in ecological traps, which entails that animals may prefer ‘less suitable habitats’ to increase survival where their fitness would be the opportunity cost [79]. However, we could get sufficient data on hunting activities as well as levels of predators during the period the presence only data was collected and suggest the need for further inquiry.

The probability distribution of elephant in 2006 was mostly influenced by distance to rivers. Similar findings where elephants preferred riverine ecosystems were reported in the Sebungwe region in Zimbabwe [41]. Like buffalo, elephants prefer utilizing riverine ecosystems for shade, and high quality browse [12, 36]. Our results showed that elephants were not influenced by vegetation cover as NDVI had little effect on their probability of occurrence. These findings are contrary to the assertion that elephants prefer greener vegetation and tend to select wooded areas and closed woodlands in the dry season [80]. However, elephants are thought to make top-down foraging decisions involving the selection of landscapes initially, then habitats within those landscapes and finally species within habitats [81]. As opposed to small isolated homogenous protected areas that may be unsuitable for elephants [80], we argue that Matetsi Safari Area benefits from the KAZA TFCA network of protected areas that offers a more heterogeneous landscape in the form of neighboring parks, e.g., Hwange, Zambezi and Chobe National Parks [82, 83].The 2014 aerial survey showed an increase in the elephant population density (i.e. 2.16 individuals per km^2^ [84]) within this region. Such high elephant densities could have been the result of migrations occurring within the KAZA TFCA and also wide-spread artificial water provisioning in the study area.

African elephant habitat suitability could not be modeled successfully using surface water availability, habitat quality and human presence for the year 2010. Other factors could have influenced the spatial distribution of African elephants in 2010 compared to 2006 such as fire. We checked the Fire Information for Resource Management System (FIRMS) for 2006 and 2010 (https://www.firms.modaps.eosdis.nasa.gov), we found that the occurrences of fire were different and as such could have contributed to the variation in the probability of occurrence of both large and medium-sized herbivores recorded in this study. Fire has been observed to influence vegetation structure and composition of ecosystems which further influence the temporal and spatial distribution of wild herbivores [85, 86]. Water provisioning may, however, reduce heterogeneity of the habitats thus making the whole landscape more suitable especially for elephants given that normally water availability is the most constraining factor in their habitat selection[36, 87].

The probability of occurrence of the medium-sized herbivores, Greater kudu and sable in this study were greatly influenced by distance from roads. Our findings confirm our expectations that human presence through road network may influence the spatial distribution of these species. Previous studies have shown that gregarious antelope species, which are commonly hunted in this area, may adjust their behavior and habitat selection in response to elevated hunting risk or exposure to human presence [33, 88]. Human activities (e.g. hunting)have been reported to shape the landscape of fear of these herbivores [89], thus we argue that roads may reduce the sense of security and cover from perceived hunting risk. Comparable to this assertion, sables occurring in the Matetsi Safari Area were observed to prefer closed woodland compared to the open habitats that they would normally occupy in the absence of disturbances associated with trophy hunting [90]. Nonetheless, greater kudu were observed to refer habitats close to roads in 2006. We argue that roads in some cases may provide unique microhabitats especially post fire episodes which are associated with re-sprouting leaves desirable for feed [91]. This is possible because in most cases, roads are burnt as fire guards as part of fire management plans in protected areas.

Surface water availability did not affect the occurrence of greater kudu in the present study, which is consistent with other findings that kudu as browsers exhibit neutrality towards water supply as they are not water dependent [92]. However, occurrence of kudu declined with increasing distance from the river. Our findings concur with other studies where kudu favored riverine ecosystem due to the availability of cover and better quality browse[36].Sable probability of occurrence was influenced by distance to artificial water points as well as NDVI. Similarly, access to surface water during the dry season has been reported to have an effect on the habitat selection by sable [93]. However, sable is sensitive to predation and competition [94]as such may avoid areas with predators and prefer to travel several kilometers to and from water points as an avoidance mechanism [95]. Rainfall has been observed as another factor that influence productivity of ecosystems and consequently species distribution [96]. For this study area, the rainfall received for 2006 (881.05 mm) and 2010 (726.30 mm) were above the long-term average rainfall (605 mm) of this region [50]. Thus, the variability in the probability of occurrence recorded for 2006 and 2010 could therefore not be attributed to rainfall variability in the study area. Further studies on the vegetation structure and composition along riverine as well as surrounding water points and the interactive effects of predation levels, trophy hunting pressure (kill sites) and fire occurrence need to be done to ensure sustainable management of these protected areas.

We recorded no evidence showing that habitat suitability for large herbivores was higher compared to medium sized herbivores in the dry season for the years 2006 and 2010 in Matetsi Safari Area. Our results are contrary to the hypothesis that large herbivores use larger proportions of the landscape and that they would be more evenly distributed than smaller herbivores due to feed quality tolerance [8]. Manipulation of surface water supply through establishment and maintenance of artificial water points may transform landscape patterns of productivity and ultimately distributional patterns of herbivore species [8]. Some management initiatives, e.g., mismanagement of fire, water provisioning, may homogenize hitherto heterogeneous landscapes [97], thus reducing the proportion of suitable habitats for such specialist grazers such as sable.

Although the species may have exhibited different distribution patterns in 2006 and 2010, the predicted total area for the large herbivores and medium-sized herbivores observed in this study was not different. Our findings are contrary to the Bell-Jarman- principle which asserts that due to tolerance of low quality diet, larger herbivores tend to use more diverse habitats compared to smaller herbivores [28]. However, there was a temporal variation in the habitat selection by both medium and large herbivores as observed for the years 2006 and 2010. We argue that though spatial separation between large and medium-sized herbivores exists in natural ecosystems, the degree of human manipulation to the ecosystem may upset these natural distribution patterns [8, 98] as also recorded in this study. Our results show that MaxEnt modeling offers an opportunity for protected area managers to model the spatial and temporal distribution of wildlife species with presence only data to aid in conservation planning and wildlife management.

## Conclusions

We predicted that surface water availability, vegetation cover and roads would have a strong influence on the spatial distribution of wild herbivores, and that habitat suitability for large herbivores would be higher compared to medium-sized herbivores in the dry season. Our findings show that large herbivores are mostly influenced by vegetation cover and distance from rivers whereas medium-sized herbivores are affected by water availability and distance from roads. We did not record any difference in the area of preferred habitats between large herbivores and medium-sized herbivores. Therefore, we conclude that: (1) habitat suitability of large and medium-sized herbivores in the dry season is mostly influenced by surface water availability, proximity to riverine ecosystems and human disturbances in the form of roads and that these factors are dynamic and highly variable thus can change from one year to another, and (2) in human mediated ecosystems (i.e. trophy hunting areas where water provisioning is practiced),large herbivores may not necessarily have larger ranging options over medium-sized herbivores due to the habitat homogenizing effect of water provisioning and associated human disturbances. We recommend that adaptive management initiatives in protected areas consider the temporal closure and or opening of water points to retain the occurrence of heterogeneous wildlife habitats in semi arid savanna ecosystems.

## Supporting Information

S1 File Presence only data for four wild herbivores collected using road counts for the years 2006 and 2010. (TXT)

S1 Table The coefficient of determination (R^2^) and the variance inflation factor (VIF) of the four environmental variables used in the model. (DOCX)
