# Supplementary material for: Habitat Heterogeneity Variably Influences Habitat Selection by Wild Herbivores in a Semi-Arid Tropical Savanna Ecosystem
Source: PLoS One. 2016 Sep 28;11(9):e0163084. doi: 10.1371/journal.pone.0163084 (PMC5040439; doi:10.1371/journal.pone.0163084)
Supplement: S1 Table — (DOCX) [file pone.0163084.s002.docx]

**Supplementary Table 1**: The coefficient of determination (R^2^) and the variance inflation factor (VIF) of the four environmental variables used in the model.

| Environmental Variable | R^2^ | Variance Inflation Factor (VIF) |
| --- | --- | --- |
| Distance to roads *vs.* distance to rivers | 0.00 | 0.00 |
| Distance to roads *vs.* NDVI | 0.02 | 0.98 |
| Distance to roads *vs.* distance to water | 0.06 | 0.94 |
| Distance to rivers *vs.* NDVI | 0.03 | 0.97 |
| Distance to rivers *vs.* distance to water | 0.01 | 0.99 |
| Distance to water *vs.* NDVI | 0.01 | 0.99 |
